# Emotion Recognition Using EEG Signals through the Design of a Dry Electrode Based on the Combination of Type 2 Fuzzy Sets and Deep Convolutional Graph Networks

**DOI:** 10.3390/biomimetics9090562

**Published:** 2024-09-18

**Authors:** Shokoufeh Mounesi Rad, Sebelan Danishvar

**Affiliations:** 1 Department of Biomedical Engineering, Urmia Branch, Islamic Azad University, 5756151818 Urmia, Iran; 2 College of Engineering, Design and Physical Sciences, Brunel University London, Uxbridge UB8 3PH, UK

**Keywords:** CNN, dry electrode, deep learning networks, emotion, graph theory, EEG, recognition

## Abstract

Emotion is an intricate cognitive state that, when identified, can serve as a crucial component of the brain–computer interface. This study examines the identification of two categories of positive and negative emotions through the development and implementation of a dry electrode electroencephalogram (EEG). To achieve this objective, a dry EEG electrode is created using the silver-copper sintering technique, which is assessed through Scanning Electron Microscope (SEM) and Energy Dispersive X-ray Analysis (EDXA) evaluations. Subsequently, a database is generated utilizing the designated electrode, which is based on the musical stimulus. The collected data are fed into an improved deep network for automatic feature selection/extraction and classification. The deep network architecture is structured by combining type 2 fuzzy sets (FT2) and deep convolutional graph networks. The fabricated electrode demonstrated superior performance, efficiency, and affordability compared to other electrodes (both wet and dry) in this study. Furthermore, the dry EEG electrode was examined in noisy environments and demonstrated robust resistance across a diverse range of Signal-To-Noise ratios (SNRs). Furthermore, the proposed model achieved a classification accuracy of 99% for distinguishing between positive and negative emotions, an improvement of approximately 2% over previous studies. The manufactured dry EEG electrode is very economical and cost-effective in terms of manufacturing costs when compared to recent studies. The proposed deep network, combined with the fabricated dry EEG electrode, can be used in real-time applications for long-term recordings that do not require gel.

## 1. Introduction

Emotions can be thought of as a complex mental state that influences human physical behaviors and physiological processes [1]. Emotion recognition has the potential to be very useful in the field of BCI [2]. This issue bridges the gap between humans and intelligent devices, allowing them to monitor changes in human emotions and mitigate their negative effects on mental health [3]. Emotions are typically divided into two categories: discrete models and dimensional models. The discrete model recognizes six different emotional states, including anger, disgust, fear, happiness, sadness, and surprise, all of which are represented by facial expressions. Additionally, the dimensional model includes valence, arousal, and neutral dimensions [4]. Arousal represents the degree of emotional activation, whereas valence represents the positive or negative emotion [5,6].

Music has been used to elicit emotions since ancient times. However, it is difficult to predict what emotions music evokes in a person. Furthermore, there are numerous methods for eliciting emotions, including words, images, sounds, and videos. However, music profoundly affects people’s emotional states and is recognized as an exceptional tool for evoking emotions while also modulating neurophysiological processes. Compared to other stimuli, music has the ability to elicit deeper and more stable emotional responses in people. As a result, the stimulation in this study is primarily musical [7].

Emotions can be recognized in two ways: non-physiological and physiological. Non-physiological signals include tone of voice, body posture, facial movement, and others of a similar nature. The aforementioned signals can be mentally controlled and hidden, so they are associated with classification errors. In contrast, physiological signals include EEG, temperature, electrocardiogram (ECG), electromyogram (EMG), galvanic skin response (GSR), and respiration [8]. EEG is a non-invasive physiological signal that directly measures the electrical activity of the brain during various emotional states [8]. These signals have several advantages over other physiological signals. EEG has several advantages, including better time resolution, faster data collection and transfer, availability, and low registration costs. EEG signals can accurately measure the spontaneous signals produced by the human brain, which include various types of emotions [8]. Despite their advantages, EEG signals have limitations. The electrodes used for Bio potentials recording have low signal-to-noise and limited spatial resolution. On this basis, distinguishing emotions from EEG signals is a difficult and time-consuming task [9]. This is why, in recent years, many models have been developed to automatically recognize emotions.

Bio potentials are recorded using different types of electrodes, which can be categorized into two groups based on their method of connection: wet electrodes and dry electrodes [10]. Prior to connection, wet electrodes require the application of several substances, such as conductive gel, to ensure effective electrode connection. However, these materials can also provide challenges for both the patient and the operator. One of the issues that needs to be addressed is the requirement to rectify the body skin or cleanse the specific region after capturing the signal [11,12]. These issues were absent with dry electrodes, which are highly favored for their user-friendly nature for both the patient and the operator. One of the obstacles in designing and using these electrodes is the electrical resistance caused by the dryness of the joint.

Bioelectrical event monitoring is a widely used procedure in therapeutic environments, and it supplies the necessary medical data for clinical and research purposes [13]. Electrophysiological measurements are employed to diagnose and assess the functionality of different organs and systems inside the body, including the heart, muscles, and nervous system. Furthermore, it is extensively mandated in operating rooms and acute care units. The bio-electrode plays a crucial role in electrophysiology. Therefore, improvements in electrode recording techniques will have a direct impact on the performance of medical systems in terms of signal quality, recording time, and patient comfort [14,15].

Many recent studies have created dry and wet electrodes for EEG recordings. However, during long-term recordings, the wet electrodes dry out and must be washed to remove the gel. Also, among the limitations of previous studies is the lack of an effective dry electrode that does not cause skin sensitivity during long-term recordings. Furthermore, the SNR ratio of dry electrodes used in recent studies is extremely low, indicating that they lack the required reliability for EEG recording. Other available electrodes include gold and other materials that are not economically viable. 

Furthermore, there is a perceived lack of a learning model capable of processing recorded EEG signals and automatically performing feature selection/extraction and classification. This study aims to overcome the aforementioned challenges and distinguish positive and negative emotions with high accuracy. This paper presents a design technique for dry bio-electrodes using copper-based silver sintering technology. This methodology encompasses all components of a precise electrode. The primary aim of this study is to fulfill the design standards for long-term portable EEG recordings. This involves optimizing the electrical performance of the electrodes by maximizing the surface area of contact between the electrodes and the skin. Furthermore, it is imperative to eliminate the mechanical, physiological, and economic constraints associated with electrode design. The proposed electrode design aims to improve the performance of existing recording systems, specifically for long-term recording devices.

This study’s contributions can be summarized as follows:

1. The design and manufacture of an effective dry electrode for long-term recording of EEG signals.
2. An EEG database based on music stimulation will be created using the proposed dry electrode.
3. A customized architecture based on the combination of FT2 sets and deep convolutional graph networks will be presented for the automatic recognition of emotions.
4. Achieving the best performance in the classification of positive and negative emotional classes compared to recent research.

The remainder of this article is written in the following manner: The second section looks at recent research on EEG bio-electrodes and automatic emotion recognition. The third section discusses the mathematical foundation used in this research. The fourth section describes the primary pipeline of this research for dry electrode fabrication, as well as the design of the proposed deep architecture. The fifth section shows the results of electrode fabrication and simulation of the proposed deep architecture. The sixth section relates to the conclusion.

## 2. Related Works

This section has two different subsections. The first sub-section examines previous research in the field of emotion recognition, while the second sub-section looks at recent research in dry electrode design.

### 2.1. Recent Research in the Field of Automatic Recognition of Emotions

Sheykhivand et al. [16] introduced an innovative, intelligent approach for detecting emotions using EEG signals. The researchers proposed a database centered around musical stimulation and successfully extracted three emotions: positive, negative, and neutral. Their proposed deep network integrated long short-term memory (LSTM) and convolutional neural networks (CNN). The proposed architecture consisted of 10 convolutional layers and three LSTM layers. Point classification was performed using two fully connected (FC) layers. The researchers attained a 97% level of accuracy by utilizing 12 channels of EEG signals. An advantage of this research was the proposed model’s ability to be used effectively in real-time environments. Baradaran et al. [17] introduced a novel model that utilizes EEG data to accurately identify emotions. The researchers proposed a model that utilized convolutional neural networks to differentiate between three emotions: positive, negative, and neutral. The study concluded with an accuracy rate of around 98%. An advantage of this research is its low computational complexity, which has resulted in an increased speed of classification. Baradaran et al. [18] introduced a novel deep model that utilizes a combination of type 2 fuzzy networks and deep convolutional networks to accurately identify three distinct emotional states. The integration of fuzzy networks alongside deep learning networks rendered the proposed model exceptionally resilient to environmental noise.

Furthermore, Generative Adversarial Networks (GAN) were employed in this study to increase the amount of data prior to its input into the deep network. The study concluded with a final accuracy rate of 98%. Yang et al. [19] employed the DEAP and SEED databases for the automated identification of emotions. One of the pre-processing steps undertaken in this study involved reducing the number of electrodes used for recording and extracting spectral features. For this study, the most effective characteristics from the recorded signals were chosen using two advanced neural networks called STCN and STCN. Furthermore, the AM-softmax classifier was employed to categorize the feature vector. The study concluded with an accuracy rate of approximately 95%. Hussain et al. [20] conducted a study on the automated identification of emotions using EEG signals. In their investigation, these scholars recorded EEG signals from 32 participants. Data preprocessing techniques used in this study included data augmentation and Z-normalization. The network used in this study was the LP–Vandy–CNN, which could automatically extract optimal features from EEG signals. The network’s classifier was also based on softmax. The accuracy achieved in this study is reported to be around 98%. 

Khubani et al. [21] used DEAP and SEED-AV databases to automatically recognize emotions. The DEaP and SEED-AV databases used in this study included 32 and 15 participants, respectively. These researchers pre-processed the data using discrete wavelet transform (DWT), statistical, and spectral analysis. The deep network proposed in this study could distinguish between different emotions with 97% accuracy. Peng et al. [22] used the DEAP database to identify emotions from EEG signals. The database used in this study included 32 participants. After obtaining the EEG signals, pre-processing, such as signal conversion from 1D to 2D and principal component analysis, were performed on the recorded signals. These researchers proposed a model for feature selection and classification using temporal relative (TR) encoding. In this study, the scores were also categorized using the softmax function. The researchers reported a final accuracy of approximately 95%. Xu et al. [23] proposed an innovative model for emotion recognition based on graph theory. The researchers conducted a study to investigate the correlation between music and brain networks. They analyzed EEG signals from 29 participants exposed to different music conditions. One advantage of the research is that it elucidates the correlation between music and the human brain, while one of its primary disadvantages is its low classification accuracy. 

Alotaibi et al. [24] utilized EEG signals for the purpose of discerning emotions. The researchers carried out their investigation using the DEAP and SEED databases. The study involved pre-processing one of the databases by applying the short-time Fourier transform (STFT). The utilized model employed pre-trained Google Net networks. In addition, emotions were categorized using the nearest-neighbors method. The researchers ultimately achieved a 96% level of accuracy. Qiao et al. [25] introduced an innovative model that uses musical stimulation to classify different emotional states. The investigators utilized a pre-existing database to extract characteristics of differential entropy. Subsequently, the CNN-SA-BiLSTM network was employed to automate the processes of feature selection/extraction and classification. The researchers reported a classification accuracy of approximately 93% for categorizing four distinct emotional classes.

Although many studies have been conducted in the field of emotion recognition using EEG signals, there are still numerous limitations that must be addressed. The limitations of previous studies include the following: none of the previous studies used dry electrodes to record EEG signals, rendering them useless for real-time applications. Because the gel related to wet electrodes dries up in long-term recordings and will reduce the SNR. Furthermore, no comprehensive database of emotions is derived from EEG signals stimulated with music. Also, the algorithms used in previous studies are computationally complex and not appropriate for real-time applications. Moreover, these algorithms are not particularly stable in noisy environments. This research is designed to overcome the limitations of previous studies, with the goal of addressing the aforementioned challenges. As mentioned in the previous section, the proposed method is based on a combination of FT2 sets and deep convolutional graph networks that can perform feature learning and classification tasks automatically. Using a combination of a combination of FT2 sets and deep convolutional graph networks makes the network extremely resistant to environmental noises and allows the model to distinguish different emotions with greater accuracy.

### 2.2. Recent Research in the Field of Dry Electrode Design and Manufacturing

Murat et al. [26] introduced a versatile dry electrode for capturing physiological signals. The researchers utilized their dry electrodes to quantify ECG. One of the benefits of this research is its ability to withstand noise. Nevertheless, in order to diminish the noise, the electrodes they devised necessitated greater pressure on the thorax, potentially causing annoyance to the patient. Jiang et al. [27] developed a coil-shaped electrode utilizing silver nanowires. The researchers utilized the performance of their electrodes to record physiological signals. Their electrode design exhibited satisfactory performance in short-term recordings; however, it induced skin sensitivity in patients during long-term registrations. Gong et al. [28] introduced an electrode made of stainless steel that is considered to be the most efficient. The electrode’s performance was utilized for the recording of physiological signals. This electrode exhibited optimal performance in recording the signal. Nevertheless, it was not financially feasible. Krachunov et al. [29] and his colleagues developed a 3D-printed electrode capable of measuring EEG signals. One of the benefits of this research was the ability to customize and provide real-time results. Wizo et al. [30] presented a new dry electrode for recording EEG signals. These researchers used an electrode to record EEG signals while sleeping. They proposed an electrode with a higher signal-to-noise ratio than the other electrode and the ability to record the signal for a longer period of time.

Tong et al. [31] demonstrated an electrode for recording EEG signals that was created using a 3D printer. To record the signal, these researchers created an electrode made of conductive material with a resistance of less than 550 ohms. The findings of this study demonstrated that alpha rhythm can be identified using the electrode proposed by these researchers. Wang et al. [32] presented a claw electrode for recording EEG signals. These researchers’ proposed electrodes would address the issue of superficial skin infections while also improving signal-to-noise ratios. Goh et al. [33] demonstrated two portable dry electrodes for recording EEG signals. These researchers assessed the performance of their research to detect alpha rhythm on a hat using a steady-state visual evoked potential and a virtual reality environment, achieving an 87% classification accuracy. Previous research has revealed the following limitations in dry electrode manufacturing: Most electrodes used in long-term applications caused skin sensitivities in participants. Furthermore, many fabricated electrodes have a low SNR ratio, which reduces classification accuracy. Also, almost all of the electrodes developed in previous studies are not economically viable. This study proposes a dry electrode for recording EEG signals that overcomes all of the limitations of previous studies.

## 3. Materials and Methods

This section examines the mathematical foundation related to the algorithms used in designing customized deep architecture for emotion recognition.

### 3.1. Brief of Graph Convolutional Network

Machine learning, a subfield of artificial intelligence (AI), enables systems and computers to acquire knowledge from data and enhance their performance without requiring specific programming for individual tasks. Machine learning finds extensive utility in several domains such as Multiplexing Enhanced Mobile Broadband (eMBB) [34], human activity recognition [35], auditory attention detection [36], person identification [37], and derivatives in Algebra [38,39].

Deep learning networks are a subset of machine learning where models are designed based on artificial neural network structures and are used to solve complex problems, including image, audio, and language processing. These networks consist of several neural layers, with each layer processing the data and extracting more features from the data. Due to the large number of layers and non-linear operations, these networks can learn more complex patterns from the data.

Graph Convolutional Networks are a type of deep neural network designed to work with data represented as graphs. Unlike traditional data, such as images or text that have regular structures (such as pixel grids or sequences), graph data are irregular and represented as nodes (nodes) and edges (edges). These types of data include social networks, chemical structures of molecules, transportation networks, and knowledge graphs. In 2016, Michael DeFerrard and his colleagues initially introduced the fundamental idea of convolutional graph networks [40]. These researchers were the first to introduce the concept of signal processing to graphs and graph spectral theory. Graph theory has facilitated the derivation of convolution functions within this field. The adjacency and degree matrices play a crucial role when applying graph theory to convolutional networks. This theory utilizes an adjacency matrix to establish connections between each vertex in the graph. The degree matrix is obtained by incorporating the adjacency matrix. Furthermore, the diagonal elements in this matrix correspond precisely to the edges that are connected to the vertex of the matrix. The degree matrix and the graph matrix are denoted as $D\in R^{N\times N}$ and $W\in R^{N\times N}$, respectively. The diagonal element of the degree matrix can be defined as follows:

(1) $$D_{\mathrm{ii}}=\sum_{i}W_{ij}$$

In this regard, the Laplacian matrix can be expressed as follows:

(2) $$L=D-W\in R^{N\times N}$$

(3) $$L=U\Lambda U^{T}$$

Based on the provided relationships, it can be inferred that the Laplacian matrix is derived by subtracting the degree matrix from the adjacency matrix. The Laplacian matrix utilizes singular value decomposition to calculate the base functions of the graph, which are then used to determine the corresponding matrix [40]. Moreover, the Laplacian matrix can be defined by establishing a connection between it and the matrices of eigenvectors and singular values. The eigenvectors of the Laplacian matrix are represented by the columns of the eigenvector matrix. The computation of the Fourier transform can be achieved by utilizing these vectors, and the introduction of the Fourier bases can be accomplished by incorporating the diagonal eigenvalues ($\Lambda =diag([\lambda_{0,\ldots ,}\lambda_{N-1}])$) into the following relationship:

(4) $$U=[u_{0},\ldots ,u_{N-1}]\in R^{N\times N}$$

To put it simply, the Fourier transform and the inverse-Fourier transform of an arbitrary signal can be defined as follows:

(5) $$\hat{q}=U^{T}q$$

(6) $$q=UU^{T}q=U\hat{q}$$

The above relations show the Fourier transform of the graph ($\hat{q}$) and the feature vector for a signal like q with Fourier bases and the Fourier transform of the graph, respectively. The deviation of the graph can be calculated by having the deviation of two signals in the domain of the graph by the Fourier transform of the signal. For example, the convolution of two signals *x* and *z*, along with the corresponding operator (${*}_{g}$), is shown below:

(7) $$x{*}_{g}=U((U^{T}x)⊙(U^{T}z))$$

where ⊙ symbolizes the element-wise Hadamard product and is calculated between the graph Fourier transformed signals. The graph convolution operator, in combination with neural networks, is described by the g() filter function. The following x-filtered relation by g(L) is displayed:

(8) $$x{*}_{g}=U((U^{T}x)⊙(U^{T}z))$$

The graph convolution can be defined using the Laplacian matrix and its decomposition into singular values and eigenvectors as follows [40,41]:

(9) $$\begin{array}{l} y=g(L)z=Ug(\Lambda )U^{T}z\\ =U(g(\Lambda ))⊙(U^{T}z)\\ =U(U^{T}(U_{g}(\Lambda )))⊙(U^{T}z)\\ =z{*}_{g}(Ug(\Lambda )) \end{array}$$

### 3.2. Brief of Type 2 Fuzzy Sets

In 1975, Professor Zadeh proposed type 2 fuzzy (FT2) sets as a continuation of type 1 fuzzy sets (FT1) [42]. In contrast to FT1 systems, FT2 systems employ membership functions that possess fuzzy membership degrees. FT2 membership functions have greatly enhanced the capacity of fuzzy systems to handle uncertainties, such as structural and measurement noise, in comparison to conventional fuzzy systems that use FT1 functions.

The ability of FT2 systems has been utilized in various research studies for designing control systems, predicting time series, and performing calculations involving words with high levels of uncertainty and complexity. The effectiveness of this ability has been demonstrated in both theoretical and practical applications. Activation functions are a crucial aspect of DNN as they significantly impact the learning process. Since the discovery of the Relu function, which is currently the most commonly used activation unit, DNNs have made significant advancements. Relu not only addresses the issue of gradient elimination but also enhances learning performance. Several activation functions, such as Relu and Leaky–Relu, have been suggested to enhance the learning performance of DNNs. Although these activation functions demonstrate good performance in DNNs, it is important to note that the input and output relationships between them are non-linear, which is a shared limitation among all of these activation functions [42,43].

In this study, the membership functions of the FT2 activator were employed in the middle layers of the proposed architecture, replacing the Relu and Leaky–Relu activation functions. This decision was based on the demonstrated capability of FT2 sets. Consequently, the proportion of its functions in the proposed network is determined as follows:

(10) $$f\left(\sigma ;\gamma \right)=\left\{\begin{array}{l} P\sigma k(\sigma ),\;\mathrm{if}\;\sigma >0\\ N\sigma (-\sigma ),\;\mathrm{if}\;\sigma \leq 0 \end{array}\right.$$

The parameter *P* controls the slope of the function in the positive quadrant, while the parameter *N* controls the slope of the function in the negative quadrant. The design parameters *γ* = [*α*, *P*, *N*] of FT2 can be seen as hyperparameters to be set or as parameters to be learned to increase the learning performance of DNNs. The *k* function can be expressed as follows [42]:

(11) $$k\left(\sigma \right)=\frac{1}{2}\left(\frac{1}{\alpha +\sigma -\alpha \sigma }+\frac{-1+\alpha }{-1+\alpha \sigma }\right)$$

If we furnish the necessary mathematical derivatives regarding the aforementioned parameters, these parameters can be employed as learning parameters. Put simply, the parameters need to be updated in every iteration, and their updating algorithm is represented by the following equations:

(12) $$\frac{\partial L}{\partial \gamma_{C}}=\sum_{j}\frac{\partial L}{\partial f_{c}(\sigma_{cj})}\frac{\partial f_{c}(\sigma_{cj})}{\partial \gamma_{c}}$$

In this context, the variable *c* denotes the layers, *j* represents the observation element, and *L* signifies the objective function of the DNN. Furthermore, $\frac{\partial L}{\partial f_{c}(\sigma_{cj})}$ symbolizes the diffusion gradient originating from the underlying layers subsequent to the FT2 phase activator layer. Its overall gradient is equivalent to:

(13) $$\frac{\partial f_{c}(\sigma_{c})}{\partial a_{c}}=\left\{\begin{array}{l} \frac{p_{c}\sigma_{c}}{2}(\frac{1}{\alpha_{c}\sigma -1}+\frac{\sigma_{c}-1}{{\left(a_{c}+\sigma_{c}-\alpha_{c}\sigma_{c}\right)}^{2}}+\frac{\sigma_{c}(1-a_{c})}{{\left(a_{c}\sigma_{c}-1\right)}^{2}})\\ \mathrm{if}\;\sigma_{c}>0\\ -\frac{N_{c}\sigma_{c}}{2}(\frac{1}{\alpha_{c}\sigma +1}+\frac{\sigma_{c}+1}{{\left(a_{c}-\sigma_{c}+\alpha_{c}\sigma_{c}\right)}^{2}}+\frac{\sigma_{c}(1-a_{c})}{{\left(a_{c}\sigma_{c}+1\right)}^{2}}\\ \mathrm{if}\;\sigma_{c}\leq 0 \end{array}\right.$$

Furthermore:

(14) $$\begin{array}{l} \frac{\partial f_{c}(\sigma_{c})}{\partial P_{C}}=\left\{\begin{array}{l} \sigma_{c}k_{c}(\sigma_{c}),\;\mathrm{if}\;\sigma_{c}>0\\ 0,\;\mathrm{if}\;\sigma_{c}\leq 0 \end{array}\right.\\ \frac{\partial f_{c}(\sigma_{c})}{\partial N_{C}}=\left\{\begin{array}{l} 0,\;\mathrm{if}\;\sigma_{c}>0\\ \sigma_{c}k_{c}(-\sigma_{c}),\;\mathrm{if}\;\sigma_{c}\leq 0 \end{array}\right. \end{array}$$

In the given equation, the value of $k_{c}(.)$ is derived using the law of updating the parameters in the specified format:

(15) $$\Delta \gamma =\rho \Delta \gamma +\xi \frac{\partial L}{\partial \gamma }$$

The ρ parameter represents the degree of movement, while the ξ parameter represents the rate of training [42,43].

Given that the FT2 activation function allows for the learning/adjustment of only *2C* parameters (*C* represents the number of hidden units), this number is relatively small when compared to the total number of weights in a normal deep neural network. In this research, the combination of FT2 activator functions and convolutional graph networks has been utilized to address uncertainties and measurement noise based on the proposed advantages of the former.

## 4. Proposed Model

This section comprises multiple distinct subsections. The following section will provide a comprehensive description of the process and design of the proposed dry electrode for EEG recording. Next, the subsequent section will provide an explanation of the database gathered using the dry electrode to measure both positive and negative emotions induced by musical stimulation. The third subsection introduces a novel, deep customized architecture for feature selection/extraction and automatic classification of two emotion classes. The fourth subsection will cover in-depth customization of architectural optimization.

**Figure 1:**
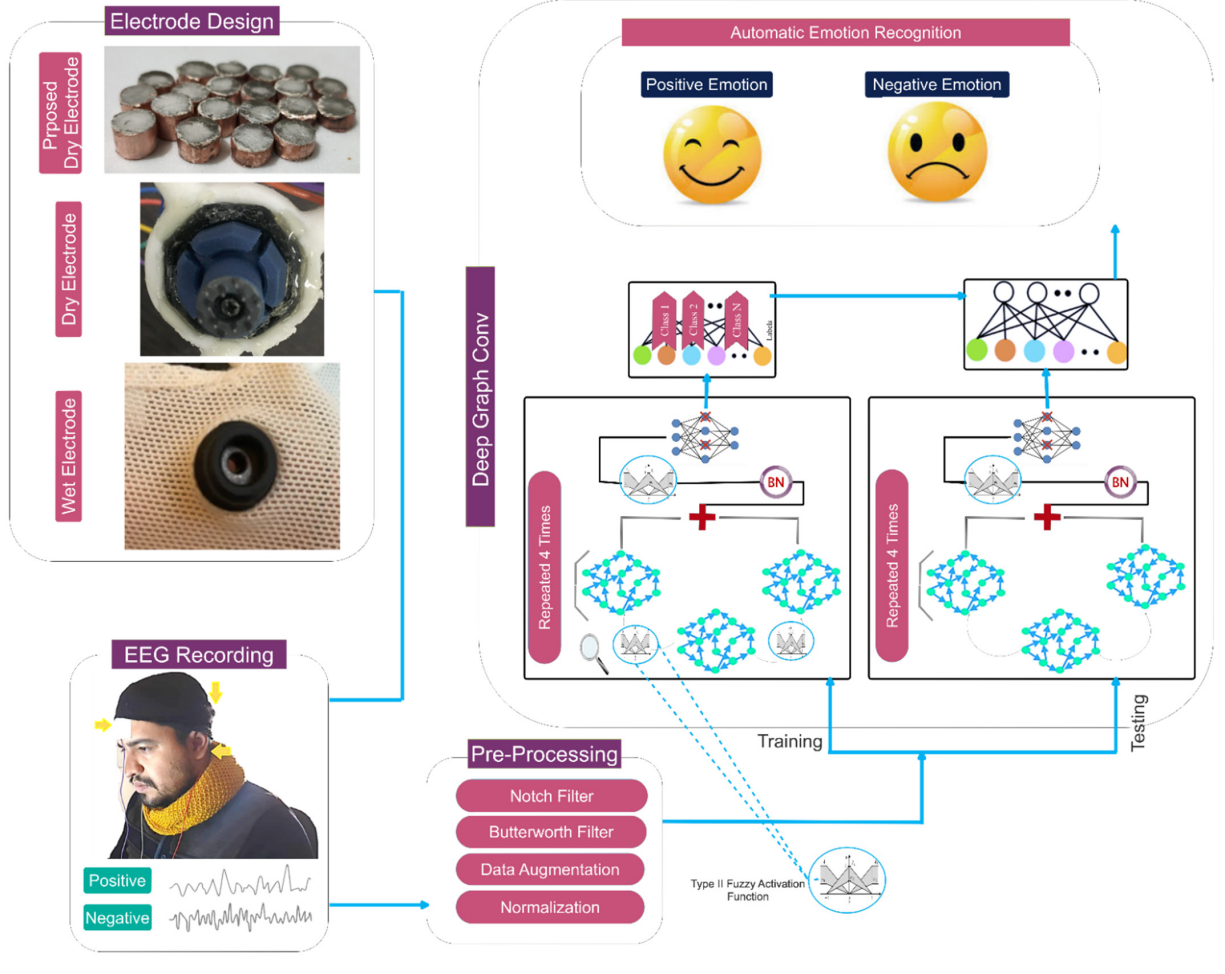
The proposed electrode design and customized deep architecture provide a general framework for classifying two types of emotions: positive and negative.

Figure 1 visually illustrates the primary structure of this study, which is based on the mentioned cases.

### 4.1. Construction and Design of Dry Electrode

**Figure 2:**
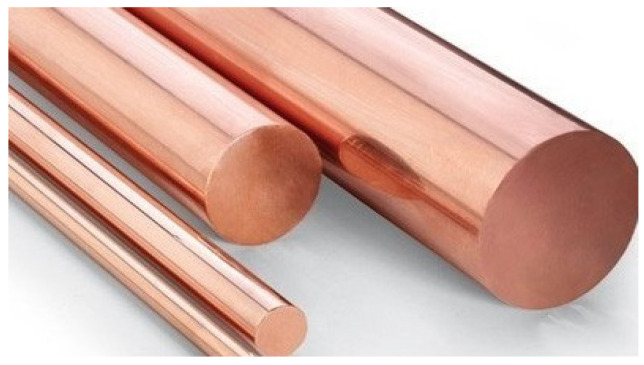
Copper bars of various diameters.

**Figure 3:**
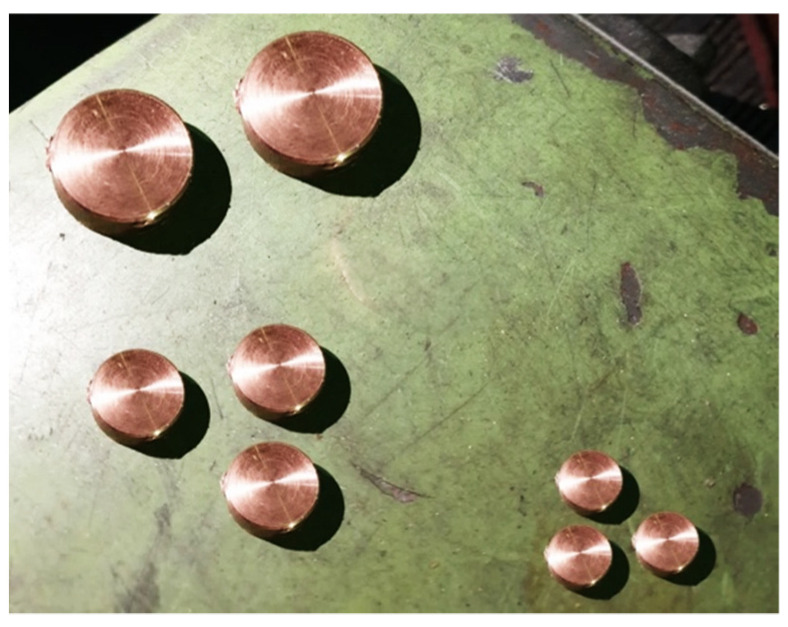
Electrode copper bases are machined and ready for sintering.

According to the explanations in the previous part on electrode production methods and materials employed by recent researchers, our proposed method for producing the electrode is based on copper and silver powder. First, the rebars seen in Figure 2 were made in various diameters for machining and cutting. The quality of the rebars employed in this investigation is thought to be 99%, resulting in good conductivity. Copper bars with 99% purity were prepared in three diameters: 10, 20, and 30 mm. To fully evaluate the performance of the samples made from these rebars, various thicknesses were chosen. Each rebar is cut into thicknesses of 4, 6, and 8 mm, resulting in nine different samples. Figure 3 shows the various thicknesses that were selected. The metal piece was cut at a temperature of less than 10 degrees Celsius. When the temperature exceeds 10 degrees Celsius, the metal surface oxidizes faster, and the copper oxide layer acts as an insulator, causing problems with the electrode’s electrical conductivity. A lathe pen was used for cutting at a low speed with air cooling. A polypropylene fiber filter was used to ensure that the compressed air was clean. Currently, silver metal powder is used to reduce the resistance of connecting the electrode to the skin while also creating a comb pattern in the microstate. Creating a connection between two different types of metal requires different methods; in this study, silver powder sintering on a copper base is used.

**Figure 4:**
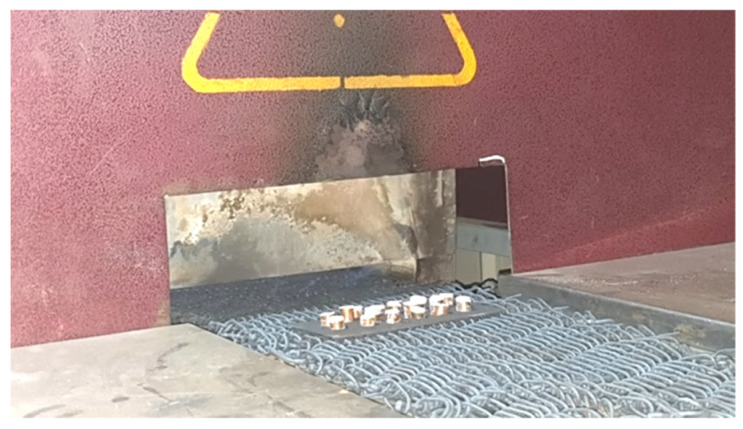
Powdered samples inside the sintering furnace.

**Figure 5:**
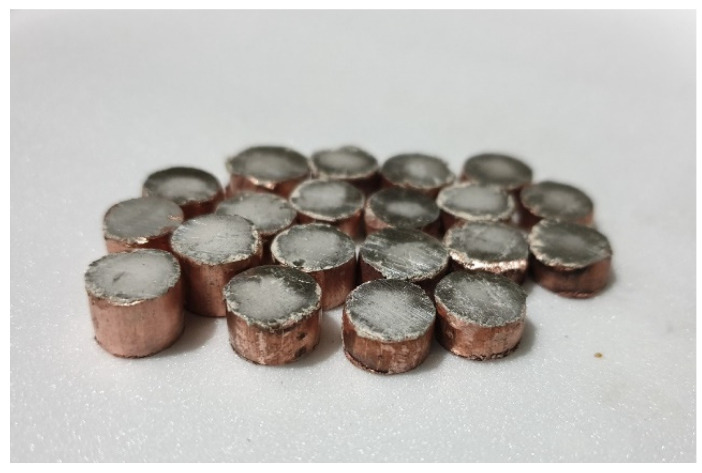
Samples taken from the furnace with a copper base and silver top.

Sintering is carried out using an automatic induction furnace, with temperature control provided by a platinum thermostat. This furnace operates in two stages. In the first, pressurized air is combined with municipal gas and heated inside the cracker. The desired temperature is achieved in the second part of the main furnace through the combustion of air and injected gas, as well as the use of electric elements. Now, the samples with different diameters and heights are ready to be powdered and placed in the furnace. A powdering device is used to ensure uniformity and control over the amount of powder on the surface. This device applies a powder layer with a thickness of 0.4 mm to the surface of the parts with great precision. Following powdering, the components are placed on an iron tray. Due to the furnace’s high temperature, graphite powder is used to prevent adhesion between the copper and the tray. In addition, the tray’s surface is coated in graphite powder. Figure 4 shows a tray with a number of samples placed on the oven’s moving chain. This process has been repeated several times with different samples and conditions to achieve the best results. Figure 5 depicts samples taken from the furnace, where the silver layer is formed as a block of zinc on a copper substrate. The mechanical destruction test between the silver layer and the copper base will reveal acceptable adhesion in the manufactured electrode.

### 4.2. Data Collection

In this section, the method of collecting EEG data using the proposed electrode will be described. The recording of EEG signals was carried out with the approval of Tabriz University’s Ethics Committee No. IR.1403.3.12 For this purpose, 20 participants (10 men and 10 women) aged 19 to 33 were chosen. The database collection comprised two distinct categories of emotions: positive and negative. During the testing phase, the researchers utilized a paper-based version of the nine-grade Self-Assessment Manikin (SAM) evaluator test to measure both positive and negative emotions. A score below 3 was deemed as low, whereas a score above 6 was regarded as high. Throughout the signal recording process, every participant in the experiment granted informed consent. Participants were given the opportunity to withdraw from the experiment at any point if they did not desire to proceed. Furthermore, all participants had a clean medical record and were instructed to refrain from consuming medication, alcoholic and energy drinks, and caffeinated beverages for a period of 72 h before the test. Furthermore, the participants were instructed to abstain from using hair conditioner and to bathe before the test. All the registrations were taken in the early hours of the morning so that the participants did not feel tired.

**Figure 6:**
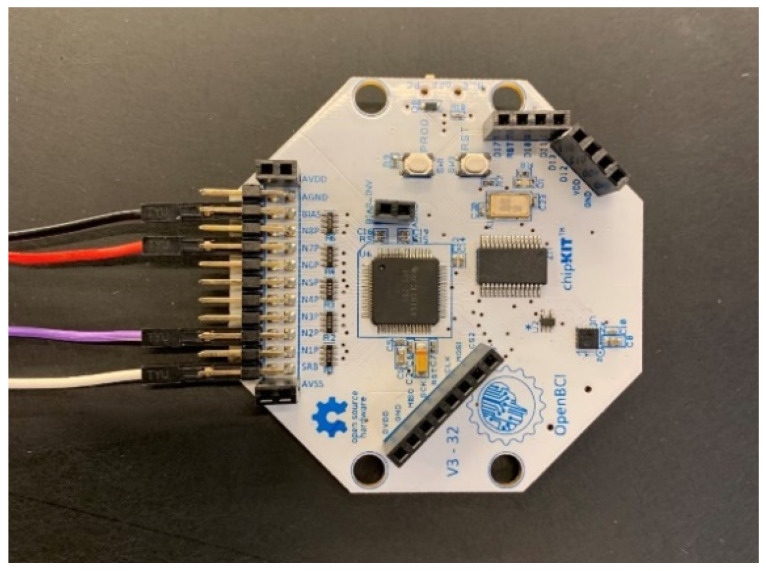
The amplifier used in the experiment for the proposed dry electrode.

**Figure 7:**
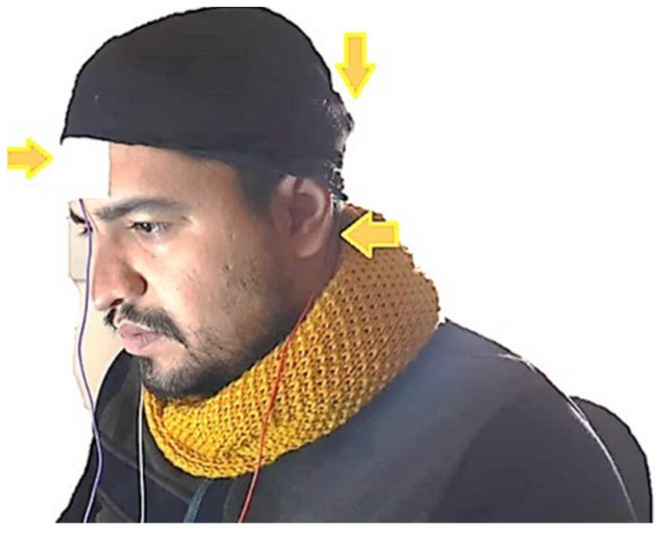
Recording of EEG signals from one of the participants based on the dry electrode (Three electrodes FP1, PZ, and FZ have been used for recording according to the image).

EEG signals were recorded using the designed electrode and a portable OpenBCI amplifier. Figure 6 depicts the amplifier used. In order to determine performance, in addition to the proposed built electrode, two other electrodes, a dry Ag/AgCl electrode from the OpenBCI brand and an Ag/AgCl coated wet electrode from the same brand, were compared to the built electrode for registration. These electrodes are shown in Figure 1. In this way, the scenarios of recording signals from participants to record emotions were performed three times using the manufactured electrodes, a wet electrode of the Open BCI brand and a dry electrode of the Open BCI brand. All registrations followed the 10–20 standard using the bipolar method. The sampling frequency for recording is 1024 Hz, and channel A1 serves as a reference. In this study, only three channels (FP_1_, P_Z_, and F_Z_) were considered for signal recording and processing, and emotion recognition was done solely based on their positions. Figure 7 displays one of the individuals involved in the experiment.

**Figure 8:**
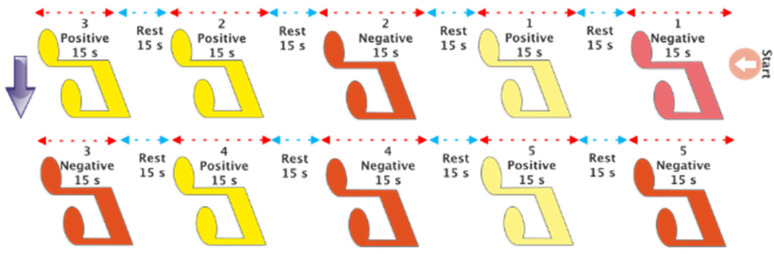
Musical stimulation scenario to evoke positive and negative emotions.

Music has been utilized to evoke both favorable and unfavorable emotions in individuals. Each musical composition was performed for a duration of 15 s with the intention of evoking both positive and negative emotions in each participant. In addition, a 15 s period of silence was incorporated between each piece in order to establish a state of neutrality. Headphones are employed for music playback in order to reduce noise interference for the EEG signal recording apparatus. Table 1 presents detailed information about the selected music for each specific emotion. According to this table, the music played for the participants included five happy songs and five sad songs. Based on the research [44], these songs were chosen to elicit both positive and negative emotions. Moreover, Figure 8 depicts the method by which the music was executed for the participants. From each participant, only one EEG signal recording session was performed while playing 10 pieces of music, according to Figure 8.

### 4.3. Pre-Processing of EEG Data

In this stage, the collected database will be pre-processed using three different types of electrodes: dry electrode, wet electrode, and dry electrode.

The initial stage of pre-processing involves the selection of electrodes. As mentioned earlier, augmenting the quantity of electrodes for signal recording enhances the precision and excellence of EEG signals. Nevertheless, augmenting the number of electrodes enhances computational efficiency, rendering the suggested model inappropriate for real-time applications. In this study, signal processing was conducted using only three electrodes: FP_1_, P_Z_, and F_Z_. In this way, 15 s of each emotion is selected (15 × 5), and it outputs 76,800 samples (75 × 1024 Hz) for each emotion.

In the subsequent stage, the data undergo filtration using a notch filter [45]. The main objective of this pre-processing is to eliminate the 50-Hz frequency associated with city electricity. A second-order Butterworth filter [46] is employed in the subsequent stage. The rationale for employing this filter is that EEG signals encompass valuable information within distinct frequency ranges. This filter effectively extracts valuable information from recorded signals within the frequency range of 0.5 to 60 Hz.

### 4.4. Architecture

To form a graph, the following operations must be performed. Following the determination of the functional connectivity of EEG channels, a proximity matrix is generated. This can be achieved by assessing the correlation between the channels and presenting the findings as an EEG channel connectivity matrix. A specified threshold is used to approximate the connectivity matrix and remove the network adjacency matrix. The generated graph is inputted into the recommended model, which identifies and categorizes features.

### 4.5. Customized Architecture

**Figure 9:**
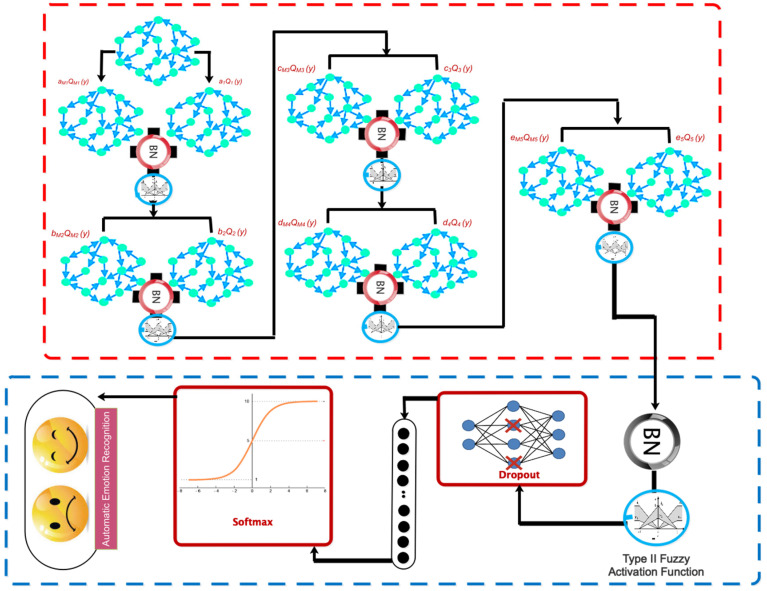
Proposed deep network representation in combination with TF2 for automatic recognition of emotions.

**Figure 10:**
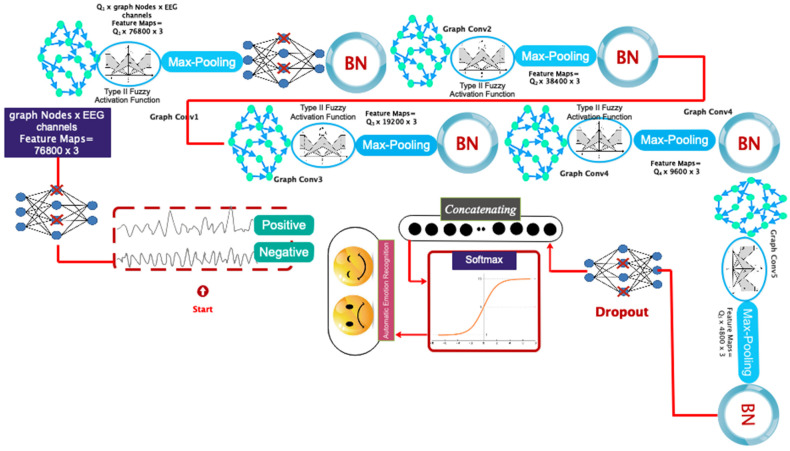
Details of each layer in the proposed pipeline.

The proposed deep model is utilized for automated emotion recognition following the construction of the graph. The proposed architecture’s overall pipeline is visually depicted in Figure 9. According to this diagram, the data are sent to a graph convolutional layer for processing after going through the dropout layer. This layer additionally incorporates a Max Pooling operation and an FT2 function. As mentioned earlier, the reason for using the FT2 function is to increase stability and deal with uncertainties such as noise. The aforementioned architecture is replicated four times without the inclusion of the dropout layer. Based on this premise, four layers of graph convolutional networks are employed to extract the dynamic information from EEG signals. Afterward, a softmax function is applied to each class to assign scores based on positive and negative excitement levels. We took the name of the described proposed network as DFCGN. Figure 10 visually represents the previously mentioned contents, including specific information about each layer. The figure indicates that the quantity of graph nodes is equivalent to the quantity of convolutional layers. Table 2 demonstrates the process of choosing the coefficients for the expansion of the Chebyshev polynomial. The selection of these coefficients in the proposed architecture is based on a process of trial and error.

Given that the SNR of the EEG signal recorded by dry electrodes is lower than that of wet electrodes, using a deep convolutional graph combination with type 2 fuzzy sets may be the best option for training the model despite noisy data. Based on this, deep convolutional graph networks, such as those used in [47], may be the most efficient method for classifying different emotions from EEG signals.

### 4.6. Training, Validation, and Test Series

The architecture of this research is structured using a trial-and-error methodology. This ensures that the parameters used in the proposed architecture are chosen optimally. The optimal parameters selected are presented in Table 3. For data training and evaluation, 70% of the dataset was allocated for network training, 20% for network validation, and the remaining 10% was reserved for network testing.

## 5. Experimental Results

This section presents the findings pertaining to the design of the proposed dry electrode and the automated identification of emotions. The initial section will showcase the outcomes of the EDEX analysis conducted on the desiccated electrode. The second part of the discussion will focus on the optimization results of the proposed architecture. The evaluation of the proposed model’s classification results will be conducted in the third subsection. The fourth subsection will provide a comparative analysis between the proposed electrode and model and other recent studies.

The deep architecture proposed in this study is implemented using the Python programming environment. The simulation results were obtained using the Google Colab platform, utilizing 32 GB RAM and a T60 graphics processing unit.

### 5.1. Optimization of the Proposed Dry Electrode

**Figure 11:**
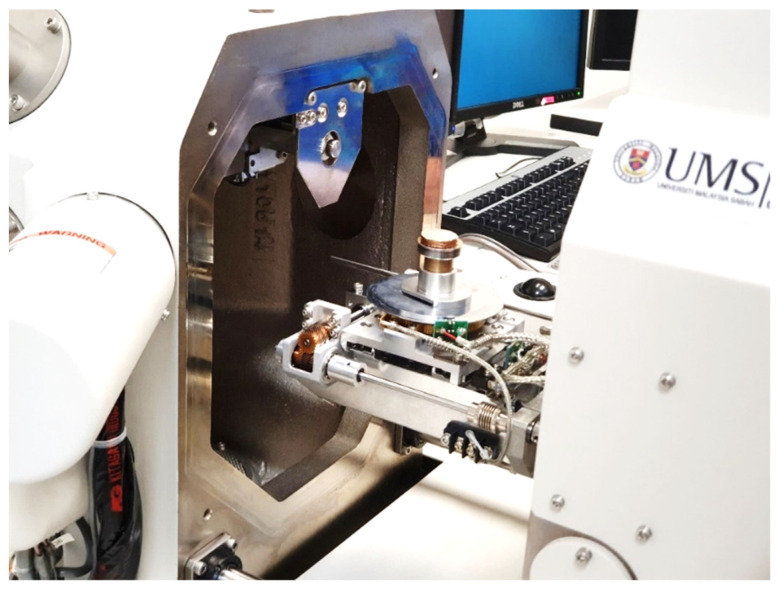
Electrode sample at the imaging point of the SEM.

This section presents the results obtained from the scanning electron microscope (SEM) for the construction of the proposed dry electrode. This microscope is one of the best tools for testing and analyzing the morphology of nanostructures, as well as identifying chemical compounds. In this study, a Hitachi brand microscope was used in the Biotechnology Laboratory of MS University Malaysia to examine the morphology of the proposed electrode. The electrode sample placed in this microscope is shown in Figure 11.

**Figure 12:**
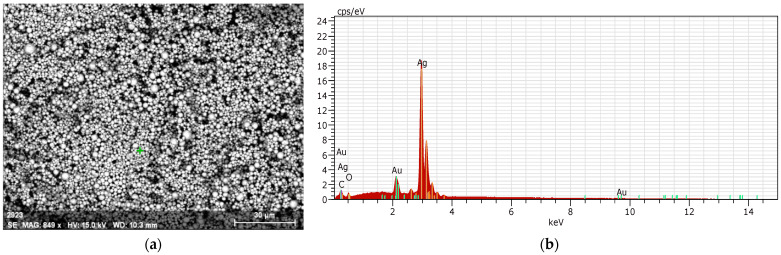
Illustrates the silver powder utilized in the annealing procedure, in conjunction with an EDXA instrument. (**a**) powder particles, (**b**) EDX results.

Figure 12 depicts the silver powder used in the annealing process, along with an Energy-Dispersive X-ray Analysis (EDXA). According to Figure 12a, the scale and size of the powder particles used are between 1 and 10 microns. Given that one of the study’s goals is cost-effectiveness, the use of this scale achieves this goal while also causing uniform sintering. If the particles are larger than 50 microns, the desired uniformity is not achieved, and the powder particles require more time and heat to form the silver block. Furthermore, in this case, the minimum cost of producing the electrode will increase tenfold and will be unaffordable. The EDX results (According to Figure 12b), as shown in this image, show the purity of the layers and the degree of metal oxidation. According to the laboratory results, the purity of silver is 86.30%, including its isotopes, and the purity of gold is 9.80%, which is due to the sample being treated and prepared with gold powder for imaging purposes. In addition, the analysis results contain 3.42% oxygen and 0.4% carbon. If the percentage of gold is ignored, the silver powder is 97% pure, with the remaining 3% attributed to silver oxide and other substances.

**Figure 13:**
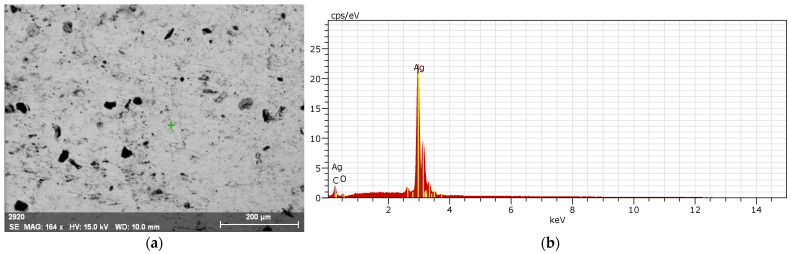
Figure 13 shows an EDXA image of the silver block that came into being on the copper base after the silver powder was sintered. (**a**) sintering of the silver powder, (**b**) EDX analysis.

Figure 13 depicts an image of the silver block formed on the copper base following the sintering of the silver powder, along with an EDXA. Figure 13a clearly shows the quality of sintering. As can be seen, there are no cracks or cracks in the powder, with only a few holes caused by the process’s low temperature in comparison to the natural melting temperature. These holes create depressions and protrusions that help to connect the electrode surface to the skin. Figure 13b depicts the EDX analysis, which, according to the laboratory report, contains 95.60% silver and its isotopes, 2.43% oxygen, and 1.98% carbon. The optimal values are achieved through precise annealing and low oxide formation at high temperatures because if the furnace’s internal atmosphere changes to a small amount and contains oxygen, the metals will be oxidized quickly due to the high temperature, and the required electrical conductivity of the electrode will not be expected regardless of the impurity.

### 5.2. Optimization of Proposed Model

**Figure 14:**
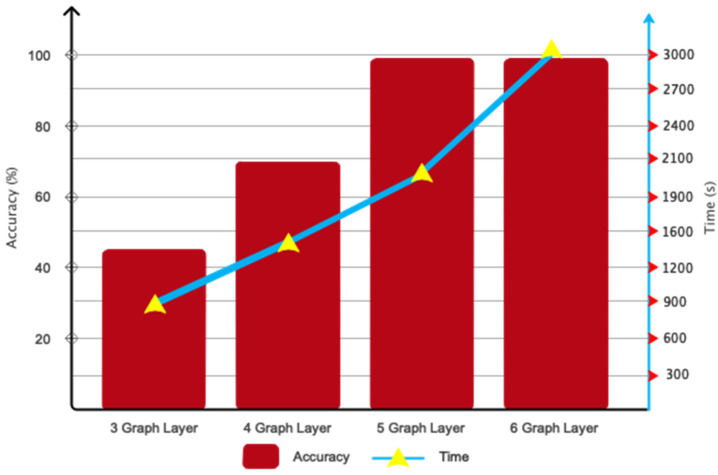
Optimization of the number and computational efficiency of the proposed DFCGN network.

**Figure 15:**
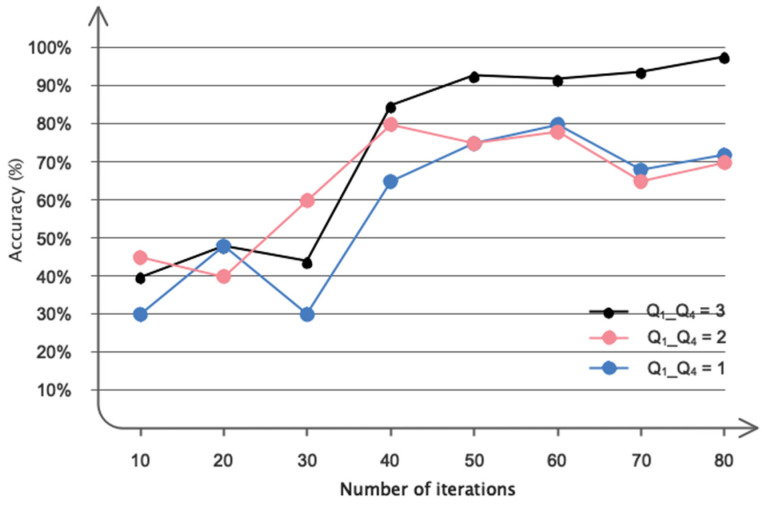
Considered polynomial values for the proposed DFCGN network.

This subsection presents the results pertaining to the optimization of the proposed architecture. Figure 14 demonstrates the significance of choosing convolutional graph layers. The data presented in this figure demonstrate that employing five convolutional layers is the optimal choice in terms of both efficiency and accuracy. Figure 15 illustrates the different Chebyshev polynomial coefficients employed in the suggested design. It is widely recognized that utilizing Q = 3 has expedited the architecture’s convergence to the desired value.

### 5.3. Results of Simulation

**Figure 16:**
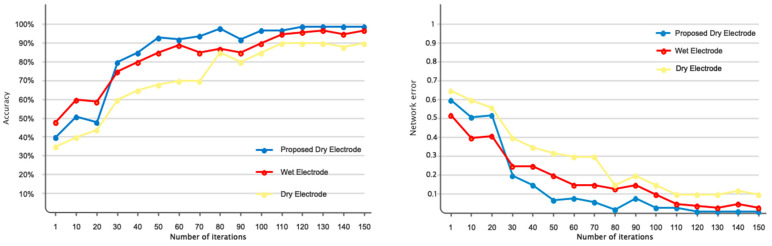
Comparison of error performance and accuracy of dry electrodes made with dry and wet electrodes from different brands. (The suggested dry electrode, dry electrode, and wet electrode are shown with blue, red, and yellow legends, respectively).

**Figure 17:**
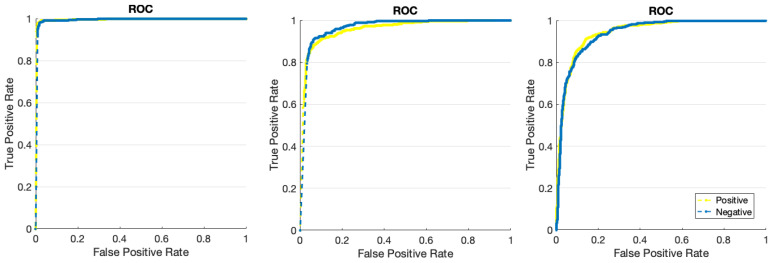
ROC diagram for the various evaluated electrodes (from left: recommended dry electrode, wet electrode, and dry electrode).

**Figure 18:**
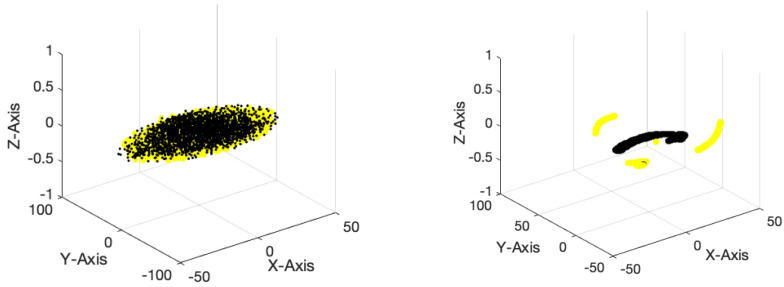
TSNE diagram for the first and last layers of the proposed DFCGN model to recognize two different classes of positive and negative emotion according to the recorded suggested dry electrode.

This section presents the simulation results of three distinct electrodes, namely the proposed dry electrode, dry electrode, and wet electrode, for the purpose of automated emotion recognition. Figure 16 displays the accuracy and error rates associated with various electrodes in the identification of two distinct categories of positive and negative emotions. The analysis was conducted using the DFCGN network, with 150 repetitions. The dry electrode based on the DFCGN network has demonstrated superior accuracy and minimal error when compared to other electrodes. Furthermore, it is evident that the electrode suggested by the deep model has demonstrated a more rapid convergence toward the target value. According to the error diagram, as the number of repetitions increases, the error rate decreases when classifying positive and negative emotions using the proposed dry electrode. Table 4 analyzes various evaluation indices using different electrodes to categorize positive and negative emotions. It is widely recognized that the proposed electrode exhibits superior efficiency when compared to other electrodes. Figure 17 depicts a Receiver operating characteristic (ROC) analysis diagram illustrating various electrodes used to categorize positive and negative emotions. The ROC diagram for the proposed electrode clearly shows that the curve falls within the 0.9 range, indicating the optimal performance of the electrode designed using the deep model. Figure 18 illustrates the positive and negative class examples for the input layer and the end layer, as determined by the deep model that was created. According to the figure, it is evident that the proposed architecture has been highly effective in segregating the samples belonging to different classes.

### 5.4. Comparison with Previous Algorithms and Studies

This subsection will provide a comprehensive analysis of the proposed model in comparison to other recent studies. Furthermore, this section will involve a comprehensive comparison and analysis of the dry electrode produced here in relation to other relevant research. Table 5 presents recent studies, along with the methodology used and the corresponding level of accuracy achieved. Based on the table, the proposed model exhibits the highest level of accuracy in comparison to recent studies. The proposed model demonstrates a precision of approximately 99% in accurately classifying two distinct categories of emotions. In contrast, the precision of studies [20,25] is approximately 98% and 96%, respectively. 

However, it would be unfair to directly compare with recent studies as they used different databases. Thus, we have employed commonly used algorithms from recent research to train our recorded data and then compared them with the results of recent studies. The algorithms employed encompass feature selection/extraction, manual classification, and feature learning. To achieve this objective, standard statistical measures such as mean, variance, skewness, kurtosis, peak coefficient, and power were derived from the data collected by the dry electrode proposed, and using Support Vector Machine (SVM) [48], Multi-Layer Perceptron (MLP) [49], K-Nearest Neighbor (KNN) [50] classifiers, The basic CNN [17] and the proposed DFCGN model were classified. Furthermore, during the second phase, feature extraction was conducted using SVM, MLP, KNN algorithms, and the proposed model, using the raw data obtained from the dry electrode. This comparative method has previously been used in studies [51,52] that are concerned with the integration of spatial, temporal, and spectral EEG signatures for predicting multilevel cognitive load and dense 3D networks based on spatial-natural focus for emotion recognition from EEG signals, respectively. The results obtained are displayed in Table 6. It is evident that employing the feature learning method with deep learning networks has enhanced classification accuracy in comparison to manual feature extraction. However, manually implementing feature extraction in deep learning networks is not appropriate. Additionally, the manual approach is highly effective in utilizing conventional classification algorithms (such as MLP, SVM and KNN). Nevertheless, employing manual techniques necessitates prior expertise in the subject or issue and has the potential to enhance the computational efficiency of the algorithm.

**Figure 19:**
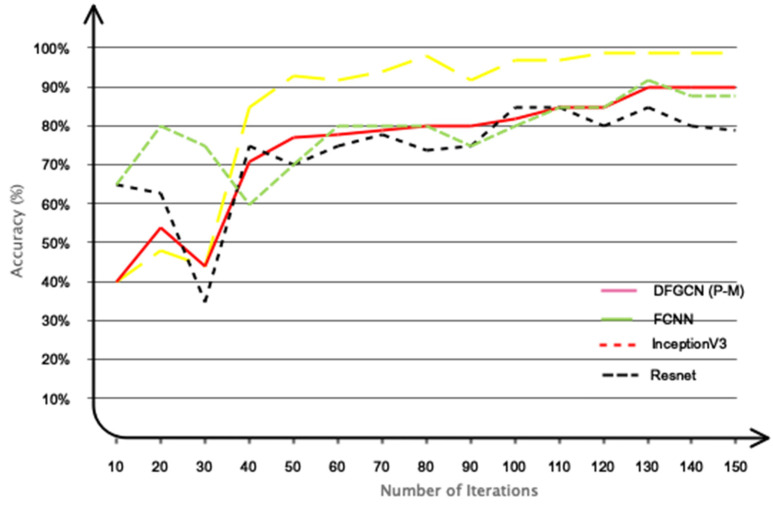
The proposed network’s performance in comparison to other networks.

Furthermore, we conducted a comparative analysis between the suggested model and other pre-trained networks that have gained significant popularity in classification research. Some of the pre-trained networks that were compared include ResNet [53], Inception [54], and FCNN. Figure 19 displays the classification outcomes obtained from the data collected using the suggested dry electrode and the deep model that was specifically developed in comparison to the pre-existing pre-trained networks. It is widely acknowledged that the proposed model exhibits superior accuracy in comparison to other networks, thereby validating the distinctive architecture of the proposed model.

**Figure 20:**
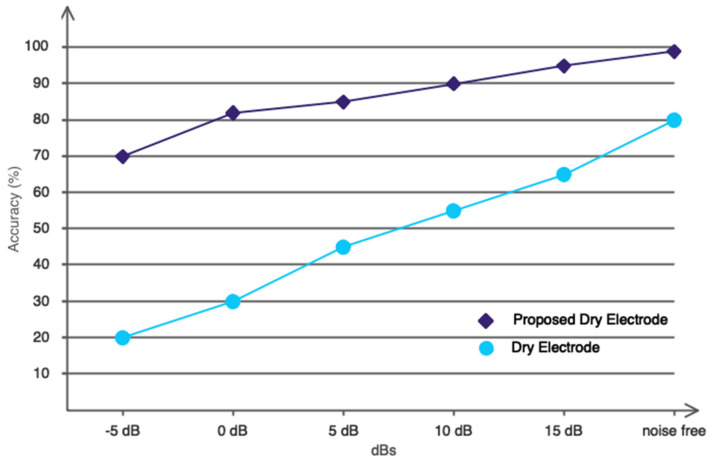
The effect of environmental noise on the proposed dry electrode and dry electrode.

As previously stated, the electrodes used to record EEG signals have a significant impact on exposure to environmental noise. Dry electrodes, especially, will have multiple effects on environmental noise due to the lack of contact gel with the skin and conductivity. Based on this, the designed electrode should be constructed in such a way that the influence of noise is significantly reduced. We considered noise in different decibels while recording EEG signals with two different types of electrodes (the proposed dry electrode and the dry electrode) [55]. Sound Meter Pro ver. 2.6.10 was used to measure the amount of noise added during signal recording, ensuring that the noise spectrum under consideration is quantitatively accurate. Figure 20 shows the classification results in different decibels after processing with the proposed DFCGN network to recognize emotions in two positive and negative classes. Based on the results, it is clear that the proposed dry electrode has a very high resistance to the noise spectrum, which can be attributed to the electrode’s unique design. As a result, the proposed dry electrode and the designed deep model make an excellent combination for emotion recognition in online applications because the built-in electrode eliminates the need to worry about the electrode gel drying or the high impact of environmental noises.

Utilizing the manufactured dry electrode for recording EEG signals in different applications can result in a cost reduction of up to 45%, making it highly cost-effective. Nevertheless, although the proposed model has shown promising results, further investigation is necessary to explore the potential use of the fabricated dry electrode in other domains, such as sleep monitoring. Furthermore, the number of emotional classes can be expanded in the existing application, allowing for a more comprehensive evaluation of the proposed deep network.

## 6. Conclusions

This study introduces a novel model for detecting positive and negative emotions. The model combines FT2 networks and deep convolutional graphs, utilizing EEG signals. In order to achieve this objective, a dry EEG electrode was specifically designed and fabricated utilizing the silver-copper sintering technique. A database was created using the constructed electrode to stimulate emotions through musical stimulation. Then, using the proposed customized deep architecture, two different classes of positive and negative emotions were classified with a high accuracy of 99%. The proposed model was evaluated against recent studies and achieved promising outcomes. The results demonstrated the feasibility of utilizing the dry electrode and the proposed deep model in real-time applications with a high level of dependability. Because of the dry electrode’s good resistance to environmental noises, it is possible to use the proposed method in real-time applications with high accuracy and reliability in brain–computer interface systems.

The present study’s limitation is the lack of evaluation in large databases. In the future, we intend to increase the number of emotion classification classes from three to nine in order to assess the performance of the developed electrode and deep model in long-term EEG recordings. Furthermore, in future studies, the constructed electrode and deep architecture can be tested and evaluated in other applications, such as sleep stage monitoring, which requires 6 h of EEG recording.

## Figures and Tables

**Table 1:** List of music performed to elicit emotions.

Emotion	N1	P1	N2	P2	P3	N3	N4	P4	N5	P5
**Music played**	Esfehani	6&8	Homayoun	6&8	6&8	Afshari	Esfehani	6&8	Dashti	6&8

**Table 2:** Details of layers, filters, etc., in the proposed pipeline.

Layer	Weight Tensor	Bias	Parameters
**GConv1**	(*Q_1_*, 76,800, 76,800)	76,800	5,898,240,000 × *Q*_1_ + 76,800
**GConv2**	(*Q_2_*, 76,800, 38,400)	38,400	2,949,120,000 × *Q*_2_ + 38,400
**Gconv3**	(*Q_3_*, 38,400, 19,200)	19,200	737,280,000 × *Q*_3_ + 19,200
**Gconv4**	(*Q_4_*, 19,200, 9600)	9600	184,320,000 × *Q*_4_ + 9600
**Gconv5**	(*Q_5_*, 9600, 4800)	4800	46,080,000 × *Q*_5_ + 4800
**Flattening Layer**	4800	2	9600

**Table 3:** Optimization of parameters in the proposed pipeline.

Parameters	Values	Optimal Value
Number of Gconv	2, 3, 4, 5, 6, 7	5
Batch Size in DFCGN	8, 16, 32	16
Batch normalization	ReLU, Leaky-ReLU, TF-2	TF-2
Learning Rate in DFCGN	0.1, 0.01, 0.001, 0.0001, 0.00001	0.0001
Dropout Rate	0.1, 0.2, 0.3	0.2
Weight of optimizer	$6\times 10^{-3},4\times 10^{-4},6\times 10^{-5},6\times 10^{-6},6\times 10^{-7}$	$6\times 10^{-6}$
Error function	MSE, Cross Entropy	Cross Entropy
Optimizer in DFCGN	Adam, SGD, Adadelta, Adamax	SGD

**Table 4:** Different evaluation indices to check the performance of the electrodes used in this study.

Measurement Index	Accuracy	Sensitivity	Precision	Specificity	Kappa Coefficient
**Proposed Dry Electrode**	99.2%	98.7%	99.4	98.4%	0.9
**Wet Electrode**	98.0%	96.4%	98.7%	99.2%	0.8
**Dry Electrode**	90.1%	88.7%	91.3%	93.8%	0.7

**Table 5:** Evaluating the proposed model in relation to recent studies.

Research	Algorithms	ACC (%)
**Sheykhivand et al. [16]**	CNN + LSTM	97
**Baradaran et al. [17]**	DCNN	98
**Baradaran et al. [18]**	Type 2 Fuzzy + CNN	98
**Yang et al. [19]**	SITCN	95
**Hussain et al. [20]**	LP-1D-CNN	98.43
**Khubani et al. [21]**	DCNN	97.12
**Peng et al. [22]**	Temporal Relative (TR) Encoding	95.58
**Xu et al. [23]**	Functional Connectivity Features	97
**Alotaibi et al. [24]**	GoogLeNet DNN	96.95
**Qiao et al. [25]**	CNN-SA-BiLSTM	96.43
**Our Model**	**New Dry Electrode + DFCGN Network**	**99.2**

**Table 6:** Comparing the use of engineering feature extraction method with feature learning to identify positive and negative emotions.

Method	Feature Learning (ACC)	Handcrafted Features (ACC)
**KNN**	65.1%	81.8%
**SVM**	72.1%	88.1%
**CNN**	92.7%	71.6%
**MLP**	70.5%	87.6%
**P-M (DFCGN)**	99.2%	78.8%

## Data Availability

The data are private and the University Ethics Committee does not allow public access to the data.
